# Cytomegalovirus Cell-Mediated Immunity: Ready for Routine Use?

**DOI:** 10.3389/ti.2023.11963

**Published:** 2023-11-07

**Authors:** Oriol Bestard, Hannah Kaminski, Lionel Couzi, Mario Fernández-Ruiz, Oriol Manuel

**Affiliations:** ^1^ Nephrology and Kidney Transplant Department, Vall Hebron University Hospital, Barcelona, Spain; ^2^ Nephrology and Kidney Transplant Research Laboratory, Vall Hebrón Institut de Recerca (VHIR), Barcelona, Spain; ^3^ Department of Nephrology, Transplantation, Dialysis and Apheresis, Centre Hospitalier Universitaire Bordeaux, Bordeaux, France; ^4^ UMR 5164-ImmunoConcEpT, University of Bordeaux, Centre National de la Recherche Scientifique (CNRS), Bordeaux University, Bordeaux, France; ^5^ Unit of Infectious Diseases, Hospital Universitario “12 de Octubre”, Instituto de Investigación Sanitaria Hospital “12 de Octubre” (imas12), Madrid, Spain; ^6^ Department of Medicine, School of Medicine, Universidad Complutense, Madrid, Spain; ^7^ Centro de Investigación Biomédica en Red de Enfermedades Infecciosas (CIBERINFEC), Instituto de Salud Carlos III (ISCIII), Madrid, Spain; ^8^ Infectious Diseases Service and Transplantation Center, Lausanne University Hospital and University of Lausanne, Lausanne, Switzerland

**Keywords:** immune monitoring, cytomegalovirus management, innate immunity, preventive strategies, antiviral prophylaxis

## Abstract

Utilizing assays that assess specific T-cell-mediated immunity against cytomegalovirus (CMV) holds the potential to enhance personalized strategies aimed at preventing and treating CMV in organ transplantation. This includes improved risk stratification during transplantation compared to relying solely on CMV serostatus, as well as determining the optimal duration of antiviral prophylaxis, deciding on antiviral therapy when asymptomatic replication occurs, and estimating the risk of recurrence. In this review, we initially provide an overlook of the current concepts into the immune control of CMV after transplantation. We then summarize the existent literature on the clinical experience of the use of immune monitoring in organ transplantation, with a particular interest on the outcomes of interventional trials. Current evidence indicates that cell-mediated immune assays are helpful in identifying patients at low risk for replication for whom preventive measures against CMV can be safely withheld. As more data accumulates from these and other clinical scenarios, it is foreseeable that these assays will likely become part of the routine clinical practice in organ transplantation.

## Introduction

Despite the implementation of effective antiviral therapies and sensitive molecular diagnostic assays, cytomegalovirus (CMV) infection remains as a major complication after solid organ transplantation (SOT), threatening both graft function and survival [1].

While relevant advances have been made in the understanding of the immunobiology of CMV infection in the context of organ transplantation, little translation to clinical practice has been done so far. In this regard, the T-cell arm of adaptive immunity (hereafter cell-mediated immunity [CMI]), especially CMV-specific CD4^+^ and CD8^+^ T lymphocytes, has been well-recognized as a major immune mechanism driving antiviral control [2, 3]. Robust evidence has showed a close association between CMV-CMI and the risk of developing CMV infection in different transplant settings [4–6]. Yet, current immune-risk stratification of CMV infection relies on the serological mismatch between donors and recipients, based on the premise that seronegative recipients receiving a seropositive graft (D+/R−) are at the highest risk of developing primary CMV infection due to their *naïve* immune status, whereas seropositive patients (R+) receiving seropositive grafts are at an intermediate risk because of previous viral immunization which should provide sufficient protection against viral replication [7]. While such paradigm has helped to predict the advent of CMV infection, this approach encompasses important limitations as a proportion of R+ individuals may unpredictably develop CMV replication and also because of the widespread use of T-cell depleting therapies that convert previously immunized patients into *naïve* individuals against CMV [8]. To minimize the development of CMV infection, the use of universal antiviral prophylaxis or preemptive assessment of viral replication are the two main preventive strategies used [7]. However, either approach is far from being accurate as they do not personalize the type and duration of such preventive strategies, since the dynamic immune status specific to CMV is not being considered.

Recently, novel immune assays have been used in transplantation showing their capacity to accurately measure CMV-CMI [4, 7]. While interesting clinical associations have been reported between CMV-CMI and the risk of CMV infection after transplant, the different methodological nature of these assays -which provide diverse biological insight on functionality of immune responses-, the so far limited data coming from clinical trials, as well as the distinct clinical transplant settings evaluated, makes it difficult to establish robust conclusions on how to implement these new technologies into clinical practice with the aim of improving transplant outcomes.

In this review, we first summarize the main mechanisms involved in the immunobiology of CMV in transplantation, to then address the major advances made with the assessment of CMV-CMI using different immune-monitoring assays as well as the major drawbacks currently limiting the implementation of these assays.

## Immunobiology of Cytomegalovirus Infection

**FIGURE 1:**
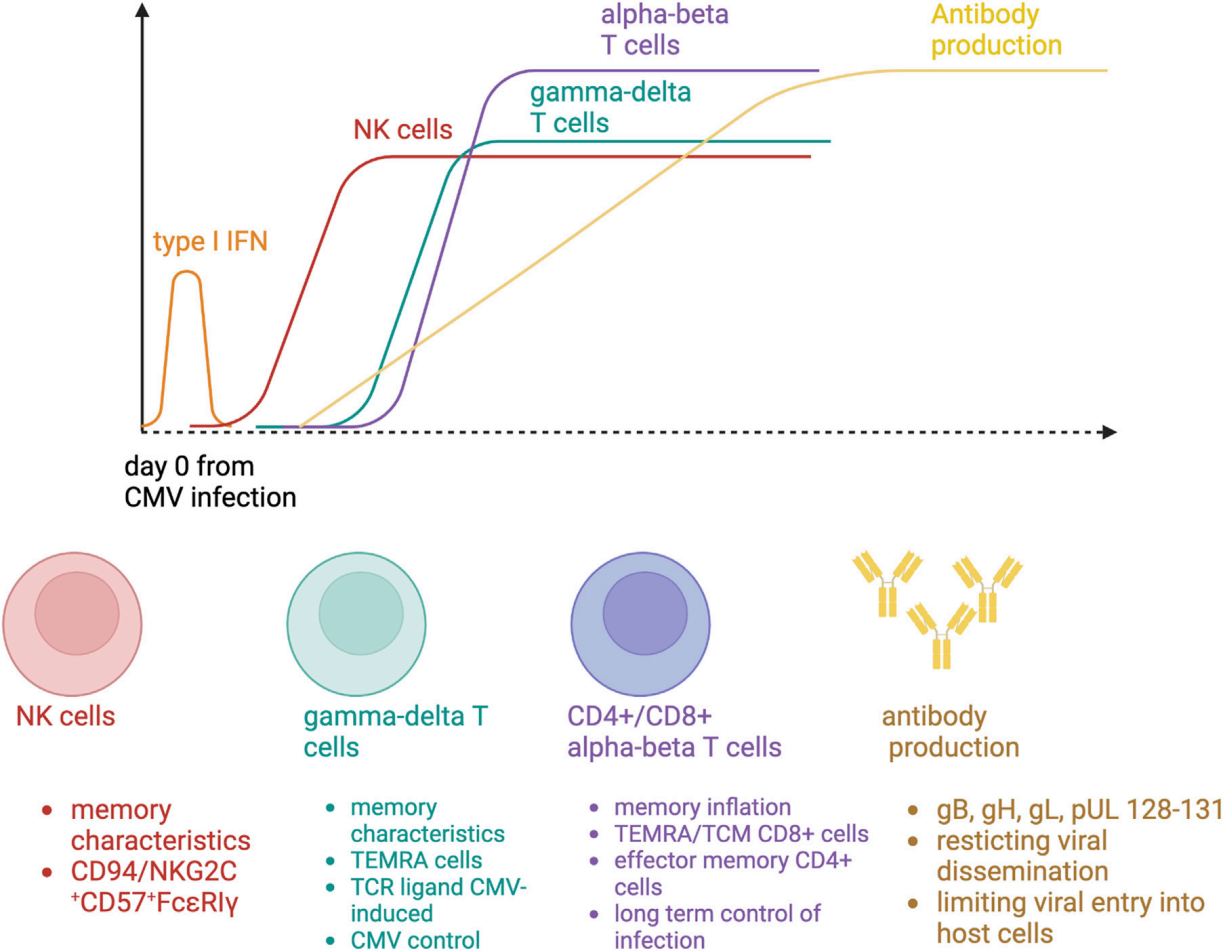
Immune responses to cytomegalovirus primary infection.

CMV infection in SOT recipients results from primoinfection or reactivation. In these two situations, a complex multi layered cell response is required to inhibit CMV dissemination [9]. Five main cell types have been studied during CMV infection, three belonging to adaptive immunity (in particular CD8^+^ and CD4^+^ T cells, and to a lesser extent the B cells) (Figure 1). Importantly, some patients do not develop CMV disease despite the absence of any CMV-specific CD8^+^ and CD4^+^ T cells, suggesting that other actors belonging to innate immunity (such as NK and γδ cells) could also be necessary for CMV control.

### NK Cells

The monitoring of NK cells can be easily performed by flow cytometry with the following fluorochrome-coupled specific antibodies: CD3, CD16, CD56, NKG2C, CD57. In human, NK cell deficiency is associated with severe herpes viral infections, such as CMV [10]. Healthy human individuals with a history of CMV infection have an expanded population of NK cells expressing the activating CD94/NKG2C receptor [11]. In kidney transplant recipients, the number of circulating NK cell is correlated with NK cell-mediated cytotoxicity during CMV infection [12]. CMV R+ patients had preexisting memory-like NK cells (NKG2C^+^CD57^+^FcεRIγ^−^) at baseline and a subset of pre-memory-like NK cells (NKG2C^+^CD57^+^FcεRIγ^low-dim^) increases during CMV DNAemia. These cells expressed a higher cytotoxic profile than preexisting memory-like NK cells at the acute phase. At later phases of viremia, a subsequent accumulation of new memory-like NK cells has been reported [13]. NK cell clonal expansion is observed after CMV infection, leading to the development of immunological memory, two features belonging to an adaptive immune response. NK cell reactivity against CMV-infected cells results from a balance governed by the activation of receptors that sense alterations in the expression of ligands on the surface of CMV-infected cells. An increase in NK activating receptors could confer to the host a better protection against CMV infection.

### *γδ* T Cells

In humans, γδ T cells are divided into two main subsets, based on their γ and δ T-cell receptor (TCR) chain expression: 1) the Vγ9Vδ2 γδT cells, expressing a δ2 chain, and 2) the non-Vγ9Vδ2 γδT cells. Initially, the involvement of non-Vγ9Vδ2 γδ T cells in the anti-CMV response was identified in the context of SOT or stem-cell transplantation. Five major observations suggest that non-Vγ9Vδ2 γδ T cells respond specifically to CMV:

- A longitudinal expansion of non-Vγ9Vδ2 γδ T cells is specifically observed in the peripheral blood of SOT recipients undergoing CMV infection [14, 15].
- CMV infection induces a restricted repertoire of non-Vγ9Vδ2 γδ T cells, suggesting an antigen-driven clonal selection [16].
- Non-Vγ9Vδ2 γδ T cells are poised for effector (particularly cytotoxic). During the course of CMV infection, non-Vγ9Vδ2 γδ T cells switch from a mainly naive phenotype (CD27^+^CD45RA^+^) towards a terminally differentiated effector memory (TEMRA) phenotype (CD27^−^CD45RA^+^), with the same kinetics than CMV-specific αβ T cells [17].
- The non-Vγ9Vδ2 T cell clones or cell lines can inhibit CMV dissemination and kill CMV-infected cells, *in vitro* [18]. Moreover, non-Vγ9Vδ2 γδ T cell expansion is associated with recovery from CMV infection without recurrence [15].
- Non-Vγ9Vδ2 T cells recognize native antigens, which are expressed at the cell surface during stress conditions (for instance CMV infection) such as reactive oxygen species (ROS) production, or AMP-activated protein kinase (AMPK)-dependent metabolic reprogramming. One example of CMV-induced γδ TCRs ligands is Annexin A2 [19].

Gamma-delta T cells can be easily monitored in clinical routine thanks to flow cytometry using a commercially available kit gathering fluorochrome coupled specific antibodies for CD45, CD3, Vδ2 and PAN-δ.

### B Cells

While the advent of long-lasting humoral immunity toward a primary viral infection is universally accepted, the contribution of antibodies for protection against and control of CMV replication in transplant recipients is still a matter of debate. Data coming from experimental models suggest a key role of B cells through CMV-specific antibody release, particularly in restricting viral dissemination and in limiting disease severity [20, 21]. CMV-specific neutralizing antibodies appear during the first 4 weeks after primary infection and are mainly directed against CMV glycoprotein B, but also H, L, and pUL128-131, all of them involved in cell attachment, penetration, and fusion of the viral envelope to the cell membrane of the host [22]. The association shown between the former use of CMV-specific immunoglobulins as prophylaxis and better outcomes among liver transplant recipients also suggests to some extend a protective role of humoral immunity against viral replication [23].

Notably, in clinical transplantation, some R+ transplant individuals remain at high risk of CMV infection despite detectable humoral immunity, suggesting either a low avidity or poor neutralizing activity of the antibody response. Post-transplant IgM and IgG antibody seroconversion has been shown not to be a reliable predictor of CMV disease [24]. Furthermore, some of D+/R− patients (20%–30%) do not develop CMV infection after transplantation, suggesting either an optimal antibody seroconversion early after transplantation or the presence of preformed CMV-specific memory B cells prior to transplantation even though undetectable circulating CMV-specific IgG antibodies [25].

### CMV-Specific CD8^+^ T Cells

During primary infection, CMV-specific CD8^+^ T cells exhibit an antigen-driven early-differentiating phenotype (CD27^+^CD28^+^ CD45RO^+^CD45RA^−^) armed for cytotoxicity [26, 27]. After viral clearance in healthy CMV R+ individuals, CMV-specific CD8^+^ T cells can represent up to 10% of the memory CD8^+^ lymphocyte pool, a process described as memory inflation [28]. There are two main subsets of CMV-specific CD8^+^ T cells: a) a central memory cell population (CD27^+^ CD28^−^ CD45RO^+^) with low cytotoxic potential but high proliferation ability, and b) a TEMRA cell population, representing up to 75% of CMV-specific CD8^+^ T cells (CD27^−^ CD28^−^ CD45RA^+^), with a low proliferation ability but a major cytotoxic potential. TEMRA cells are resupplied from central memory cells and *naive* precursors.

During primary infection, the CMV-specific CD8^+^ T cell population is polyclonal. On the opposite, few epitope‐specific clones are predominant at the chronic phase. More than half of individuals have CD8^+^ T cell recognizing CMV peptides from the three following open reading frames (UL48, UL83, UL123). UL123 (immediate-early [IE]-1)-specific CD8^+^ T cells are associated with less CMV reactivation in SOT recipients, likely because UL123 is the first CMV protein to be expressed in infected cells. *In vitro*, CMV-specific CD8^+^ T cells can kill autologous CMV-infected cells and inhibit CMV dissemination. In mouse models, late effector CD8^+^ T cells maintain long-term control of viral replication [29].

### CMV-Specific CD4^+^ T Cells

After a primary infection in SOT recipients, CMV-specific CD4^+^ T cells can be detected 1 week after the occurrence of CMV DNAemia [30], more specifically those CD4^+^ CD28-granzyme B+ cells [30, 31]. At the chronic phase of infection after viral clearance, CMV-specific CD4^+^ T cells represent up to 9% of the memory T lymphocyte pool. They exhibit an effector memory phenotype (CD27^−^ CD28^−^ CD45RA^−^). More than half of individuals have CD4^+^ T cells recognizing CMV peptides transcribed from the five following open reading frames (UL55, UL83, UL86, UL99, UL122). CD4^+^ T cells play a central role in anti-CMV immunity by clearing cells loaded with CMV peptides, helping B cells to mount a specific humoral response against viral antigens and CD8^+^ T cells to perform their effector functions [32].

### Immunosuppressive Therapy and CMV Immune Response

CMV-CMI is abrogated for one to 3 months after anti thymocyte globulin induction [8] and reduced in patients having received high-dose steroids [33]. Rejection is usually treated by these two drugs and is therefore a risk factor for CMV disease [34, 35]. *In vitro*, tacrolimus is a potent inhibitor of CMV-specific cytokines release [36], and completely inhibits activation and proliferation of CMV-specific T cells [37]. On the opposite, belatacept demonstrated minimal inhibitory effects on CMV-specific T cells likely because of an absence of effect on cells lacking CD28 [36, 37]. While the antiviral immune response against CMV measured *in vitro* appears preserved under belatacept [38], high‐risk belatacept‐treated recipients show defects in sustaining CMV control [39], and exhibit high incidence of atypical life-threatening CMV diseases [40]. Further research is needed to elucidate this gap. Finally, a dysfunctional T-cell profile (with high PD1, low CD85j expression) has been observed in CMV-infected patients receiving mycophenolic acid. On the contrary, everolimus can improve T-cell fitness and transform dysfunctional into functional cells, along with better control of CMV [41]. In summary, the analysis of these five cells types could be useful for transplant physicians to understand the impact of the immunosuppressive regimen on CMV-specific T response.

## Observational Data on the Clinical Application of CMV Immune-Monitoring

A growing number of observational studies have assessed in recent years the clinical usefulness of CMV-CMI monitoring to guide patient management in different SOT populations [42]. This research mainly includes single-center studies—with some multicenter experiences [8, 33, 43–47]— and has been performed in a wide range of clinical risk scenarios (Table 1). The most common methodologies used for the measurement of CMV-CMI is the reference technique of intracellular cytokine staining (ICS) by flow cytometry [42, 44, 45, 59, 61, 62, 67–71] and the different platforms for interferon (IFN)-γ release assay (IGRA) [4, 43, 46–56, 60, 63, 65, 66, 72–75]. Out of these immune assays, only three are currently commercially available: the quantiFERON®-CMV (QTF-CMV) (Qiagen, Hilden; Germany), the T-SPOT^®^.CMV (Oxford Immunotec, Abingdon, United Kingdom) and the T-Track®CMV (Mikrogen, Neuried, Germany). Available experience with the major histocompatibility complex (MHC)-tetramer staining method is more limited [64], whereas a few studies have compared the diagnostic accuracy of different approaches [54, 57, 76]. In most cases the primary study outcome is any CMV viremia, regardless of the presence or absence of symptoms or the level of DNAemia, or less often clinically significant viremia requiring antiviral therapy [46]. Since R+ patients typically have a low incidence of CMV disease [77, 78], the few studies that have primarily investigated the role of CMV-CMI monitoring to predict the occurrence of symptomatic infection (viral syndrome or end-organ disease) are focused on the high-risk group D+/R− patients [43, 48, 58]. Notably each platform has different readouts that are directly related to the nature of each immune assay. In general, all assays measure T-cell mediated effector immune responses of IFN-Ɣ production in response to two main immunogenic CMV antigens, phosphoprotein 65 (pp65) and IE-1 [79]. Importantly, while ELISA-based assays do not provide the individual response to each CMV antigen, flow-cytometry and ELISpot-based assays do deliver such the specific immunes, thus better illustrating the global burden of immune responses against CMV.

**TABLE 1:** Summary of observational studies assessing the potential application of CMV-CMI monitoring in different clinical scenarios.

Clinical scenario	Predicted event	Supporting studies	Monitoring method	Proposed intervention
High-risk patients (D+/R−, T-cell-depleting antibodies, lung transplantation) during antiviral prophylaxis or at the time of discontinuation	Late-onset disease^a^	Yes [43, 46, 48–58]	QTF-CMV, ELISpot	Prolong antiviral prophylaxis or close monitoring for viremia if inadequate response
Pre-transplant assessment in intermediate-risk patients (R+ with no other factors)	Post-transplant viremia and/or disease	Yes [4, 44, 47, 51, 59, 60]	QTF-CMV, ELISpot, ICS	Initiate antiviral prophylaxis or close monitoring for viremia in patients with inadequate response (D+/R_NR_)
Intermediate-risk patients (R+) on preemptive therapy with no concurrent viremia	Subsequent viremia and/or disease	Yes [42, 44, 49, 51, 52, 61–64]	ICS, QTF-CMV, ELISpot, MHC-tetramer staining	Reduce the frequency and/or discontinue monitoring of viremia if adequate response
Intermediate-risk patients (R+) on preemptive therapy with asymptomatic viremia	Spontaneous clearance	Yes [65, 66]	QTF-CMV	Withhold antiviral therapy if adequate response
Active CMV infection or disease during antiviral treatment	Response to antiviral treatment	No		Decrease immunosuppression and/or modify antivirals if inadequate response
Active CMV infection or disease after discontinuation of antiviral treatment	Post-treatment relapse	Yes [67]	ICS	Initiate secondary prophylaxis if inadequate response
Acute graft rejection treated with steroid boluses and/or T-cell-depleting antibodies	Disease following anti-rejection therapy	No		(Re)initiate prophylaxis if inadequate response

CMV, cytomegalovirus; D, donor; ELISpot, enzyme-linked immunosorbent spot assay; ICS, intracellular cytokine staining; QTF-CMV, QuantiFERON-CMV assay; MHC, major histocompatibility complex; R, recipient.

^a^ Refers to the occurrence of CMV, disease after discontinuing antiviral prophylaxis with ganciclovir or valganciclovir (usually administered for 100–200 days).

As shown in Table 1, the available literature is not equally distributed across the different clinical scenarios involved. One of the most immediate applications of CMV-CMI monitoring is the individualization of the length of prophylaxis. Rather than the fixed-duration regimen of 3–6 months of valganciclovir—up to 12 months for lung transplant recipients—recommended by the current guidelines for high-risk patients [7, 80], the knowledge of the CMV-CMI functionality would allow for prematurely discontinuing prophylaxis in patients that have mounted a protective response, or prolonging it beyond the standard schedule in the presence of a negative (non-reactive) assay result. Manuel et al. provided early data on the usefulness of the QTF-CMV assay in a multicenter cohort of 127 D+/R− patients. The presence of a positive (reactive) assay at the end of valganciclovir prophylaxis was associated with a lower 12 months incidence of CMV disease as compared to negative or indeterminate results (6.4% versus 22.2%, respectively; *p*-value < 0.001), yielding a positive predictive value (PPV) for immune protection of 90% (95% confidence interval [95% CI]: 74–98). Interestingly, those patients with an indeterminate QTF-CMV result—suggestive of a profoundly abrogated immunity or absence of CMV peptide recognition—had the highest incidence of late disease [43]. These findings have been subsequently confirmed in different SOT populations [53, 57, 75]. On the other hand, a recent study has suggested that the predictive accuracy in this clinical scenario of commercially available ELISpot assays is superior of that of the QTF-CMV assay [57]. A similar conclusion may be drawn from a meta-analysis in kidney transplant recipients [81]. The next natural step is to apply this evidence to the clinical decision-making process. In addition to the interventional studies reviewed in the next section, a retrospective study in lung transplant recipients reported a lower incidence of high-level CMV replication by using a QTF-CMV-guided strategy of extended valganciclovir prophylaxis (5–11 months) as compared to a fixed 5 months regimen (43.1% versus 60.3%, respectively; *p*-value < 0.001) [55]. These results were replicated using the T-SPOT^®^.CMV in a distinct cohort of R+ lung transplant recipients [82].

Although the ability of the QTF-CMV assay to stratify the risk of late CMV disease following the discontinuation of prophylaxis has been demonstrated for the D+/R− constellation, some studies restricted to R+ kidney transplant recipients receiving T-cell-depleting induction therapy (ATG) [54] or R+ lung transplant recipients [56] failed to find significant differences in the occurrence of viral reactivation between patients with reactive or non-reactive results. It has been proposed that the diagnostic accuracy of the QTF-CMV assay to predict protection from low-level infection among R+ patients might be improved by increasing the threshold for IFN-γ production used to define a positive result [54]. In addition, more sensitive techniques not restricted to CD8^+^ T-cell responses, such as ICS by flow cytometry and ELISpot-based assays, would perform better in this scenario, at the expense of being more time-consuming and costly [83].

The predominant population of R+ seropositive SOT recipients without ATG has been traditionally considered as an intermediate risk for CMV events, and either preemptive therapy or antiviral prophylaxis are recommended as prevention methods [7, 80]. A major contribution of the strategies for measuring the CMV-CMI has been the identification of a subgroup of R+ patients that lacks or displays very weak effective T-cell-mediated responses against CMV at the pre-transplant evaluation (non-reactive recipients [R_NR_]) despite their positive anti-CMV IgG serological status. The proportion of R+ patients with no detectable baseline CMV-CMI has been estimated at about 20%–30% [44, 59, 60, 84, 85]. From a functional perspective, these patients should be considered closer to the seronegative recipients (R−) than to the so-called intermediate-risk (R+) group, which would result in a higher susceptibility to post-transplant infection if they receive an organ from a seropositive donor [25]. In a study in kidney and lung transplant recipients, Cantisán et al. found that D+/R_NR_ patients faced a markedly increased risk of CMV replication as compared to R+ patients with a positive (reactive) pre-transplant QTF-CMV assay (adjusted odds ratio [OR]: 10.49; 95% CI: 1.88–58.46) [60]. Comparable results have been obtained with the ICS technique [44, 59] or an ELISpot assay [51, 85]. An early assessment at post-transplant day 15 provides a predictive capacity significantly higher than at the pre-transplant evaluation since some transplant recipients with robust preformed CMV-CMI may significantly decrease their functional CMV-CMI after induction immunosuppression therapy, even in absence of ATG [44]. In this regard and unlike the QTF-CMV assay, the knowledge of the specific CMV-CMI against each individual CMV antigen that is provided by ELISpot-based assays, may further help to better stratify patients according to three distinct immunological risks, this is, at low, high, and at intermediate risk if one response against one of the two antigen is absent or very low [33]. Some factors have been reported to be associated with the absence of QTF-CMV reactivity among R+ SOT candidates such as profound lymphopenia, younger age, the type of organ to be transplanted, presence of certain recipient HLA genotypes and of non-HLA-A1/non-HLA-A2 alleles [84]. The latter finding is not unexpected as the presentation to the CD8^+^ T-cells of the viral epitopes contained in the “antigen tube” of the assay is restricted through some HLA class I alleles [86, 87].

Finally, some studies have been conducted to investigate the usefulness of post-transplant CMV-CMI monitoring among intermediate-risk recipients preemptively managed to predict protection against the development of CMV infection or, once established, the capacity of spontaneous clearance of viremia [42, 44, 49, 51, 52, 61–66]. These results pointed to the predominance of CD8^+^ T-cells in the early response to primary infection—or re-infection in the D+/R+ constellation—and CD4^+^ T-cells in the long-term control of latent infection [42, 44, 61]. The assessment of CMV-CMI at the onset of asymptomatic CMV viremia may be also useful to discern the patients that will spontaneously clear the infection from those who would eventually benefit from preemptive therapy. By applying the cut-off value for QTF-CMV positivity of ≥0.2 IU/mL of IFN-γ, Lisboa et al. reported a sensitivity and specificity in this clinical scenario of 82.8% and 75.0%, respectively, yielding a negative predictive value to predict virologic and/or clinical progression of asymptomatic viremia of 54.5% and a PPV of 90.9% to predict spontaneous clearance [65].

Few observational studies have also explored the role innate cells (NK and Non-Vγ9Vδ2 γδT cells) in different scenarios. For instance, pretransplant peripheral blood NKG2C+ NKG2A- NK cells could protect from CMV infection in kidney transplant recipients independently of the presence of CMV-specific T cells [88]. The NKG2C+ NK cell proportion in the bronchoalveolar lavage could also be a relevant biomarker for assessing risk of subsequent CMV viremia in lung transplant recipients [89]. During acute CMV infection, the NKG2C+ NK cells proliferate, become NKG2C(hi), and finally acquire CD57, a marker of “memory” NK cells that have been expanded in response to infection [90]. During CMV disease, non-Vγ9Vδ2 γδT cells expansion was correlated to the resolution of CMV infection and the emergence of CMV resistance in kidney transplant recipients, but more importantly was able to predict the absence of recurrence [15, 91]. A prospective clinical trial is ongoing to confirm this last finding (SPARCKLING study: NCT03339661).

Finally, as a complement to the assessment of the functionality of the CMV-specific T-cell response, other immunological biomarkers have been proposed to improve the process of risk stratification in the SOT population. This includes the assessment of antibodies targeting the pentameric complex (gH/gL/pUL128/pUL130/pUL131A), post-transplant hypogammaglobulinemia, absolute counts of total lymphocytes or peripheral blood subpopulations, as well as genetic markers. A detailed account of the advantage and limitations of these assays is summarized in Table 2.

**TABLE 2:** Other immunological approaches proposed within the risk assessment for post-transplant CMV infection.

Immunological biomarker	Rationale	Diagnostic performance, advantages and limitations	Selected studies
Serum immunoglobulin levels	Severe IgG HGG (usually defined by the threshold of <400–500 mg/dL) as a quantitative surrogate of the humoral immune response	Easily available and economical (nephelometry). Potentially reversible by IVIg/SCIg replacement therapy. Lack of specificity for CMV infection risk	[92, 93]
Total lymphocyte count	Lymphopenia (usually defined by the threshold of <0.5–0.75 × 10^3^ cells/μL) as a quantitative surrogate of the T-cell-mediated immune response	Easily available and economical. Lack of specificity for CMV infection risk	[94–97]
Peripheral blood lymphocyte subpopulations	Enumeration of peripheral blood CD4^+^ and CD8^+^ T-cell counts at different post-transplant time points by automated flow cytometry	Less time- and labor-consuming than CMV-CMI monitoring. Lack of specificity for CMV infection risk. Simultaneous risk assessment for other opportunistic infections. Of particular usefulness in patients receiving T-cell-depleting agents	[98, 99]
SNP in genes orchestrating innate and adaptive responses (pattern recognition receptors and interferons)	Protective effect associated to SNPs within *TLR9* and *IFNL3* genes. Risk-conferring effect associated to SNPs within *TLR2, MBL2, DC-SIGN, IL10* and *IFNG* genes	Attempts of polygenic risk scores (lacking external validation). Modest risk modification effect attributable to a given SNP. Lack of dedicated GWAS studies	[100–104]
Intracellular ATP production in CD4^+^ T-cells	Quantification of intracellular ATP release in CD4^+^ T-cells stimulated with a potent non-specific mitogen (phytohemagglutinin A), which would provide an overall functional evaluation of T-cell-mediated immunity	FDA-approved commercial assay (ImmuKnow^®^, Cylex). Lack of validated cut-off values to predict CMV infection. Time- and labor-consuming. Potentially affected by sample storage time	[56, 105]

ATP, adenosine triphosphate; CMV, cytomegalovirus; CMVCMI, cytomegalovirus-specific cell-mediated immunity; FDA, food and drug administration; GWAS, genomed-wide association study; HGG, hypogammaglobulinemia; IVIg, intravenous immunoglobulin; SCIg, subcutaneous immunoglobulin; SNP, single-nucleotide polymorphism.

## Interventional Studies Evaluating CMV Immune Monitoring Strategies

The evidence generated by clinical trials on the use of CMV-CMI in transplant recipients is more limited. Most randomized controlled trials have focused on using the CMV-CMI assays for determining the duration of antiviral prophylaxis in intermediate or high-risk patients, particularly in kidney transplant recipients. In these studies, analysis of CMV-CMI has been performed using either the QTF-CMV or an ELISpot-CMV assay (Table 3).

**TABLE 3:** Summary of the intervention studies on the application of CMV-CMI assays in SOT recipients.

Study author	Number of patients	Type of organ transplant	CMV serostatus	Cell-mediated immune assay	Intervention	Main results
[106]	118	Lung	R+ and D+/R−	QTF-CMV	Test at 5, 8 and 11 months, stop prophylaxis if test positive	Lower CMV replication in the allograft and longer duration of antiviral prophylaxis in the intervention group
[107]	150	Kidney	R+ on ATG	QTF-CMV	Test at 30, 45, 60, 90 days, stop prophylaxis if test positive	Similar incidence of CMV replication/disease, shorter duration of antiviral, lower incidence of neutropenia in the intervention group
[108]	185	Kidney (164) and liver (21)	R+ on ATG and D+/R−	T-Track-CMV	Test at 30, 60, 90 days (R+ and D+/R−), 120, 150, 180 (D+/R−), stop prophylaxis if test positive	Similar incidence of CMV replication/disease, shorter duration of antiviral in the intervention group
[109]	27	All SOT	R+ and D+/R−	QTF-CMV	Test at the end of therapy for CMV replication, add secondary prophylaxis in case of negative result	Lower incidence of CMV relapse in patients with a positive test
[110]	160	Kidney	R+	T-SPOT.CMV	Stratify patients at transplant in low vs. high-risk according to test result. Then randomize to preemptive vs. prophylaxis	Higher incidence of CMV replication in high-risk group. Better performance of antiviral prophylaxis strategy in both groups

ATG, anti-thymocyte globulin; CMV, cytomegalovirus; D, donor; QTF-CMV, QuantiFERON-CMV assay; R, recipient.

In the study by [106] 118 lung transplant recipients were randomized to receive a fixed duration of antiviral prophylaxis (5 months), or a duration based on the results of the QTF-CMV assay, performed at 5, 8 and 11 months after transplantation. Antiviral prophylaxis was continued in case of a negative result of the assay in the intervention group. CMV replication measured by PCR in the bronchoalveolar lavage was observed in 58% in the control group as compared to 37% in the intervention group (*p* = 0.03), and this effect was probably due to the longer duration of prophylaxis in patients in the intervention group. A significant number of patients (39%), mostly D+/R−, remained with undetectable CMV-CMI at the end of prophylaxis period.

In the TIMOVAL trial [107], R+ kidney transplant recipients receiving induction therapy with ATG were randomized to receive a fixed duration of 3 months of prophylaxis (control group) or a duration based of immune-monitoring every 2–4 weeks using the QTF-CMV assay. Despite receiving ATG, up to 45% of patients had a QTF-CMV result as soon as 30 days after transplantation. Incidence of CMV infection (17% immune-monitoring vs. 13% control) was similar between groups while duration of antiviral prophylaxis was shorter in the intervention group. Incidence of neutropenia was lower in the immune-monitoring arm.

In the CMV-CMI study from Switzerland [108] 185 kidney or liver transplant R+ recipients receiving ATG or D+/R− were randomized to receive 3 or 6 months of prophylaxis (depending on the risk group) or immune monitoring once monthly with the T-Track-CMV^®^. Overall, the incidence of clinically significant CMV infections was similar between groups (30.9% immune-monitoring vs. 31.1% group) although non-inferiority was not proven (*p* = 0.06). The duration of antiviral prophylaxis was significantly shorter in the intervention group (−26 days, *p* < 0.001). The impact of the intervention was more pronounced in R+ patients.

Kumar et al. [109] performed a single-arm interventional study using a QTF-CMV assay at the end of antiviral therapy for clinically significant CMV infection (both CMV disease and asymptomatic replication). Patients with a positive QTF-CMV result did not receive additional antiviral therapy while patients with a negative result received valganciclovir for 8 additional weeks. Of the 27 SOT recipients included, 14 patients had detectable IFN-γ levels and 13 had undetectable levels. Only 1/14 (7%) patient with a positive assay result had a relapse of CMV replication in contrast with 9/13 (69%) in the group with a negative assay result.

Finally, in the RESPECT trial [26], Jarque et al. used the T-SPOT. CMV at the time of transplant to stratify patients as being low-risk (positive assay) or at high-risk (negative assay) based on IE-1 CMV-CMI for predicting post transplant CMV replication. Patients were then randomized to receive antiviral prophylaxis or a preemptive approach. Patients with a positive CMV-CMI test had significantly lower rates of CMV replication/disease irrespective of the preventive strategy used. However, the best performance of the assay was when performed at 15 days post transplant (81% of CMV infection if test negative vs. 9% if test positive).

Although more interventional studies would be desirable to better delineate the clinical scenarios for the use of CMV-CMI monitoring in SOT recipients, a summary of the main data available is provided below.

**FIGURE 2:**
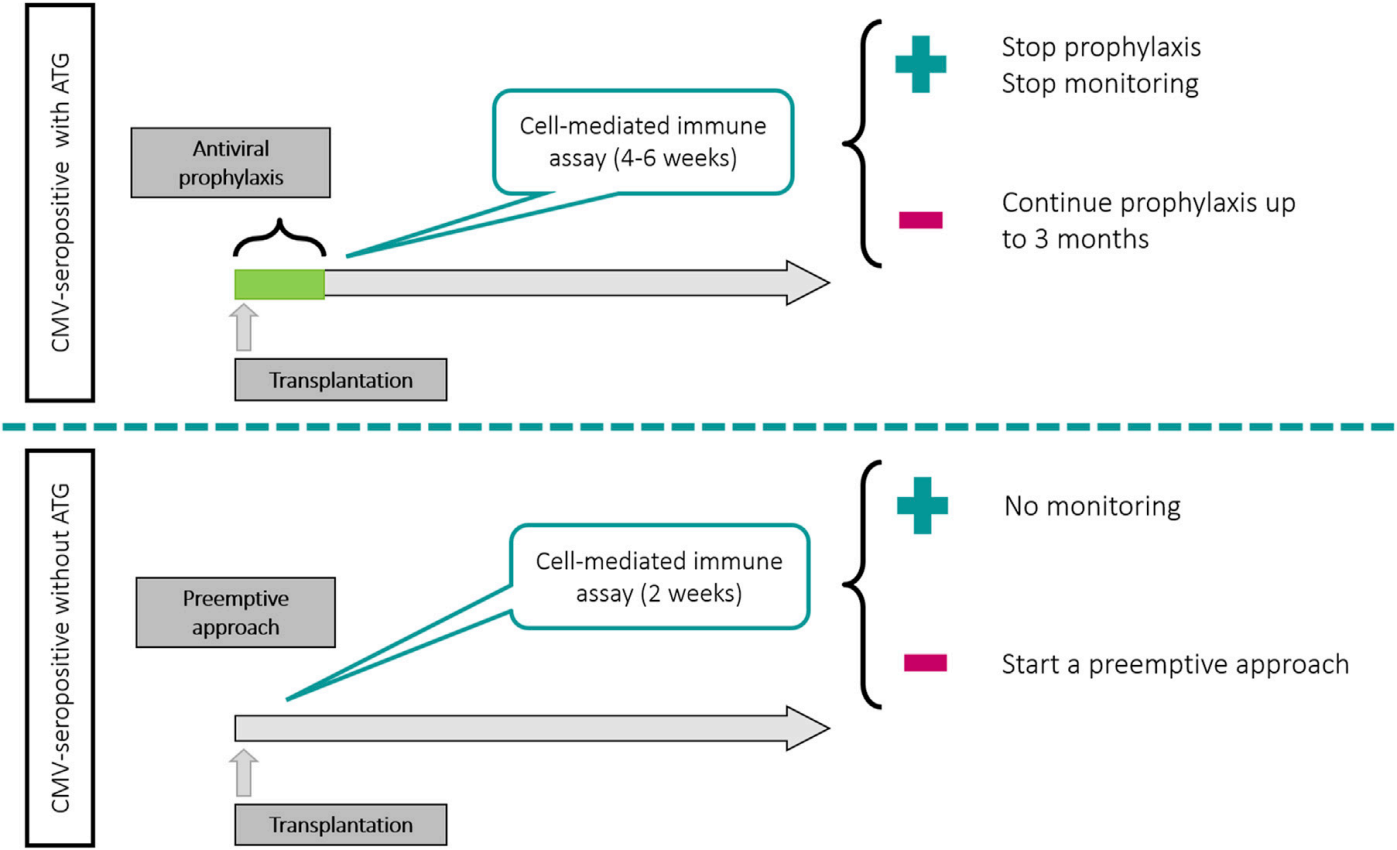
Potential uses of CMV-CMI assays in CMV-seropositive patients according to preventive strategy and use of T-cell depleting antibodies.

• A CMV-CMI assay can be used in the pre transplant period (if no T-cell depletion will be used) to identify those patients with a negative or low pre-transplant CMV-CMI and thus being at higher risk of CMV infection and therefore to choose the most appropriate preventive strategy against CMV. However, a positive CMV-CMI test prior to transplantation may lead to misleading predictive interpretations since a proportion of these patients may become high risk after transplantation due to induction immunosuppressive therapy.
• In patients receiving universal prophylaxis, the most appropriate population for using these assays seems to be the CMV-seropositive patients receiving ATG (as proposed in the TIMOVAL and CMV-CMI trials). According to these studies, as soon as 1 month post transplant, the majority of patients (45%–62%) mount a measurable CMI response against CMV, associated with a low risk for developing CMV disease. A potential strategy for these patients can be to perform a single-point assay at 4–6 weeks after transplant and to stop antivirals if the test is positive. In case of a negative result, an extension of prophylaxis or a preemptive approach could be applied. Figure 2 illustrate a potential management of R+ patients according to the use of ATG.
• In patients managed with a preemptive approach, a CMV-CMI assay could be used in CMV-seropositive patients without receiving ATG (based on the RESPECT trial [110]). Here the risk of significant CMV replication is much lower and the probability to reach a detectable immune response much higher than in patients receiving ATG. A potential strategy for these patients can be to perform a single-point assay at 2 weeks after transplant and to stop PCR monitoring if the test is positive (Figure 2).
• There is limited data for high-risk D+/R− patients. In the CMV CMI study [108], the impact of the use of CMI assays was less visible in the high-risk group, mainly because the mounting of immunity was achieved later after transplant, and in only a minority of patients. A potential strategy in this population would consist in assessing CMV-CMI between 4 and 6 months post transplant and stop prophylaxis in case of a positive assay. Given the suboptimal sensitivity of CMV-CMI assays in this population, a negative result should not foster the extension of prophylaxis, but rather a closer follow-up after discontinuation of antivirals.

## Conclusion

In this review we show the advances made in the field of CMV immune-risk stratification with the development of new sensitive assays measuring CMV-CMI. While most of the studies strongly suggest an added value of measuring CMV-CMI to better stratify the risk of CMV, in particular among R+ SOT recipients, yet some concerns arise when translating these immune tools into clinical practice; the precise predictive values illustrating the risk at the patient-individual level should be noted with caution to ultimately establish safe, guided preventive strategies. Specific cut-offs, the biological insight provided by each type of assay, and the precise clinical settings where to be implemented need to be further investigated through the implementation of clinical trials.

With the implementation of artificial intelligence, including highly powerful machine-learning algorithms, the combination of distinct clinical as well as immunological variables at distinct biological level could further refine the individual risk of transplant patients to develop CMV infection. Notably, this is the ultimate goal of the large multicenter European project (HORUS^1^) by developing a dynamic multidimensional biomarker algorithm to robustly assess the risk of developing CMV infection.

Therefore, an effort should be made among the transplant community to confirm the added value of cell-mediated immune assays over current clinical management, as though if confirmed, they could revolutionize the management of CMV infection by personalizing the type and duration of preventive therapy against CMV infection after SOT.

## Footnotes

1
https://www.horus-project.eu/
